# Diagnostic accuracy of whole-body MRI versus standard imaging pathways for metastatic disease in newly diagnosed colorectal cancer: the prospective Streamline C trial

**DOI:** 10.1016/S2468-1253(19)30056-1

**Published:** 2019-05-09

**Authors:** Stuart A Taylor, Sue Mallett, Sandy Beare, Gauraang Bhatnagar, Dominic Blunt, Peter Boavida, John Bridgewater, Caroline S Clarke, Marian Duggan, Steve Ellis, Robert Glynne-Jones, Vicky Goh, Ashley M Groves, Ayshea Hameeduddin, Sam M Janes, Edward W Johnston, Dow-Mu Koh, Anne Miles, Stephen Morris, Alison Morton, Neal Navani, John O'Donohue, Alfred Oliver, Anwar R Padhani, Helen Pardoe, Uday Patel, Shonit Punwani, Laura Quinn, Hameed Rafiee, Krystyna Reczko, Andrea G Rockall, Khawaja Shahabuddin, Harbir S Sidhu, Jonathan Teague, Mohamed A Thaha, Matthew Train, Katherine van Ree, Sanjaya Wijeyekoon, Steve Halligan, Streamline investigators, Ruth Evans, Simon Ball, Revanth Jannapureddy, Tina Mills-Baldock, Kishor Barhate, Zoltan Nagy, Sherif Raouf, Akosa Aboagye, Girija Anand, Rommel Butawan, Elizabeth Hadley, Adesewa Onajobi, Kathryn Tarver, Tanjil Nawaz, Catherine Norman, Nathalie Rich, Sidra Tulmuntaha, Shafi Ahmed, Louise Lim, Fiona McKirdy, Jenna Couture, Shahanara Ferdous, Payal Julka, Ali Mohammed, Terry O'Shaughnessy, William Ricketts, Marie Jackson, Clive Kay, Andy Lowe, Janet McGowan, Amjad Mohammed, Jon Robinson, Lara Curry, Sasithar Maheswaran, Subramanian Ramesh, Pippa Riddle, Shaki Balogun, Yvonne Campbell, Nelesh Jeyadevan, Aji Kavidasan, Imogen Locke, Tuck-Kay Loke, Ibiyemi Olaleye, Clare Collins, Elizabeth Green, Colm Prendergast, Thida Win, Amy Davis, Lyn Blakeway, Sofia Gourtsoyianni, Adrian Green, Christian Kelly-Morland, Sahar Naaseri, Davide Prezzi, David Snell, Dorothee Boisfer, Keyury Desai, Balinder Hans, Sophia Hans, Eleni Ntala, Adnam Alam, Stephen Burke, Angshu Bhowmik, Nishat Bharwani, Gule Hanid, Lesley Honeyfield, Tina Stoycheva, Nicola Strickland, Farid Bazari, Helen Beedham, Jane De Los, Reyes Lauigan, Priya Limbu, Nicola Lucas, Sally O'Connor, Anita Rhodes, Laletha Agoramoorthy, Martha Handousa, Abel Jalloh, Stefania Stegner, Shanna Wilson, David Birch, Suzanne Chukundah, Priscilla Phiri, Raj Srirajaskanthan, Eleni Karapanagiotou, Daniel Smith, Ferrial Syeed, Chloe van Someren, Rudi Borgstein, Jamila Roehrig, David Chao, Lorraine Hurl, Andrew Gogbashian, Andre Nunes, Ian Simcock, James Stirling, Richard Beable, Maureen Furneaux, Nicola Gibbons, Antony Higginson, Howard Curtis, Kitrick Perry, Anita Amadi, Heather Hughes, Prital Patel, Gary Atkin, Colin Elton, Stephen Karp, Lisa Woodrow, Dominic Yu, Sajid Khan, Alistair Rienhardt, Pooja Datt, Rajapandian Ilangovan, Ian Jenkins, Saba Mahmud, Teresa Light, Joanne Kellaway, Ann O'Callaghan, William Partridge, Amelia Daniel, Ugo Ekeowa, Michael Long, Peter Russell, Erica Scurr, Veronica Morgan, Nina Tunariu, Elizabeth Chang, Laura Hughes, Ellice Marwood, Katie Prior, Meena Reddi, Kara Sargus, Abby Sharp, Teresita Beeston, Elizabeth Isaac, Adoracion Jayme, Jagadish Kalasthry, Wivijin Piga, Farzana Rahman, Shraddha Weir, Aileen Austria, James Crosbie, Alec Engledow, Jonathan McCullogh, Austen Obichere, Kai-Keen Shiu, Christopher Wanstall, Celia Simeon, Amy Smith, Andrew Bateman, David Breen, Liane Davis, Chris Everitt, Alice Johnson, Paul Nichols, Beth Shepherd, Kayleigh Gilbert, Azmina Verjee, Michelle Saull, Jonathan Wilson, Rashidat Adeniba, Veronica Conteh, Sarah Howling, Sara Lock

**Affiliations:** a Centre for Medical Imaging, University College London, London, UK; b Cancer Research UK & UCL Cancer Trials Centre, University College London, London, UK; c Institute of Nuclear Medicine, University College London, London, UK; d Lungs for Living Research Centre, UCL Respiratory, University College London, London, UK; e Research Department of Primary Care and Population Health, University College London, London, UK; f Department of Applied Health Research, University College London, London, UK; g Institute of Applied Health Research, NIHR Birmingham Biomedical Research Centre, College of Medical and Dental Sciences, University of Birmingham, Edgbaston, Birmingham, UK; h Frimley Park Hospital, Frimley, UK; i Imaging Department, Imperial College Healthcare NHS Trust, London, UK; j Department of Radiology, Homerton Hospital, London, UK; k Department of Surgery, Homerton Hospital, London, UK; l UCL Cancer Institute, London, UK; m Department of Radiology, Barts Health NHS Trust, London, UK; n Mount Vernon Centre for Cancer Treatment, Mount Vernon Hospital, Northwood, UK; o Department of Cancer Imaging, School of Biomedical Engineering and Imaging Sciences, King's College London, King's Health Partners, London, UK; p Department of Thoracic Medicine, University College London Hospitals, UK; q Department of Radiology, Royal Marsden Hospital, Sutton, Surrey, UK; r Department of Psychological Sciences, Birkbeck University of London, London, UK; s Department of Gastroenterology, Lewisham Hospital, London, UK; t Paul Strickland Scanner Centre, Mount Vernon Cancer Centre, Northwood, UK; u Intestinal Imaging Centre, St Mark's Hospital, LNWUH NHS Trust, Harrow, UK; v Norfolk and Norwich University Hospitals NHS Foundation Trust, Norwich, UK; w Department of Radiology, Hammersmith Hospital, Imperial College Healthcare NHS Trust, London, UK; x Department of Cancer and Surgery, Imperial College London, London, UK; y Blizard Institute, National Bowel Research Centre, Barts and the London School of Medicine and Dentistry, Queen Mary University of London, London, UK; z Department of Surgery, Barts Health NHS Trust, The Royal London Hospital, London, UK; aa Department of Radiology, Royal Free London NHS Foundation Trust, London, UK

## Abstract

**Background:**

Whole-body MRI (WB-MRI) could be an alternative to multimodality staging of colorectal cancer, but its diagnostic accuracy, effect on staging times, number of tests needed, cost, and effect on treatment decisions are unknown. We aimed to prospectively compare the diagnostic accuracy and efficiency of WB-MRI-based staging pathways with standard pathways in colorectal cancer.

**Methods:**

The Streamline C trial was a prospective, multicentre trial done in 16 hospitals in England. Eligible patients were 18 years or older, with newly diagnosed colorectal cancer. Exclusion criteria were severe systemic disease, pregnancy, contraindications to MRI, or polyp cancer. Patients underwent WB-MRI, the result of which was withheld until standard staging investigations were complete and the first treatment decision made. The multidisciplinary team recorded its treatment decision based on standard investigations, then on the WB-MRI staging pathway (WB-MRI plus additional tests generated), and finally on all tests. The primary outcome was difference in per-patient sensitivity for metastases between standard and WB-MRI staging pathways against a consensus reference standard at 12 months, in the per-protocol population. Secondary outcomes were difference in per-patient specificity for metastatic disease detection between standard and WB-MRI staging pathways, differences in treatment decisions, staging efficiency (time taken, test number, and costs), and per-organ sensitivity and specificity for metastases and per-patient agreement for local T and N stage. This trial is registered with the International Standard Randomised Controlled Trial registry, number ISRCTN43958015, and is complete.

**Findings:**

Between March 26, 2013, and Aug 19, 2016, 1020 patients were screened for eligibility. 370 patients were recruited, 299 of whom completed the trial; 68 (23%) had metastasis at baseline. Pathway sensitivity was 67% (95% CI 56 to 78) for WB-MRI and 63% (51 to 74) for standard pathways, a difference in sensitivity of 4% (−5 to 13, p=0·51). No adverse events related to imaging were reported. Specificity did not differ between WB-MRI (95% [95% CI 92–97]) and standard pathways (93% [90–96], p=0·48). Agreement with the multidisciplinary team's final treatment decision was 96% for WB-MRI and 95% for the standard pathway. Time to complete staging was shorter for WB-MRI (median, 8 days [IQR 6–9]) than for the standard pathway (13 days [11–15]); a 5-day (3–7) difference. WB-MRI required fewer tests (median, one [95% CI 1 to 1]) than did standard pathways (two [2 to 2]), a difference of one (1 to 1). Mean per-patient staging costs were £216 (95% CI 211–221) for WB-MRI and £285 (260–310) for standard pathways.

**Interpretation:**

WB-MRI staging pathways have similar accuracy to standard pathways and reduce the number of tests needed, staging time, and cost.

**Funding:**

UK National Institute for Health Research.

## Introduction

Colorectal cancer is the second leading cause of cancer-related deaths in the UK, with about 16 000 deaths annually.^1^ Accurate staging is fundamental for optimal patient outcomes, particularly identification of metastatic disease, because this typically dictates therapeutic strategy. Up to 50% of patients with metastatic disease relapse after apparently curative surgery.^2^ Upfront detection of metastases would allow appropriate use of chemotherapeutic, surgical, and ablative therapies.^3^

Staging pathways are complex, relying on high technology imaging platforms such as CT, PET-CT, and MRI. In England, for example, the National Institute for Health and Care Excellence (NICE) publishes guidelines that require multiple, sequential imaging tests to complete staging and allow the first treatment decisions to be made.^4^ The complexity of staging pathways is due to modalities having variable accuracies across organs at risk for harbouring metastases. Standard pathways are, therefore, time and resource intensive, irradiate patients,^5^ and increase anxiety if they are protracted.^6^

**Research in context**

**Evidence before this study**

The detection of metastatic disease during colon cancer staging underpins treatment strategy and is fundamental to the optimisation of patient outcomes. Staging pathways rely on high technology imaging platforms such as CT, PET-CT, and MRI, which differ in their diagnostic accuracies across individual organs. Such multimodality staging pathways are complex, resource and time intensive, involve irradiation, and increase patient anxiety. Modern MRI platforms can image the whole body within 1 h, and whole-body MRI (WB-MRI) is advocated as a more accurate, efficient, and safer alternative to multimodality staging pathways. We searched PubMed and Embase (without language restriction) for articles published between Jan 1, 1990, and Sept 30, 2018, using MeSH and full-text search-strings for “cancer”, “neoplasm” “staging”, “diagnostic accuracy”, “magnetic resonance imaging”, “whole body imaging”, “diffusion magnetic resonance imaging”, “metastasis”, “colorectal”, and “colon”. We found several meta-analyses reporting WB-MRI accuracy for cancer staging. Many combined different primary cancers in single analyses or were limited to detecting metastasis in single organs (or both). Most meta-analyses compared WB-MRI with PET-CT, and scintigraphy (in the case of bone metastasis), rather than CT alone, which is the test used most commonly in colorectal cancer staging. No meta-analysis considered colorectal cancer in isolation. Most primary studies were small, single site, and explanatory, with WB-MRI interpreted by a few specialised radiologists. They focused on single modality comparisons rather than evaluating real-world, multimodality staging pathways. We found no data regarding how WB-MRI influences the first major treatment decision or staging efficiency.

**Added value of this study**

To our knowledge, this is the largest prospective multicentre trial to date comparing the diagnostic accuracy of WB-MRI staging pathways to standard staging in patients newly diagnosed with colorectal cancer. We used a pragmatic trial design to better test pathway performance in routine clinical practice and investigated pathway efficiency in terms of test number, time to completion, and costs. We also contemporaneously tested the effect of alternative staging pathways on the nature and timing of the first major treatment decisions. Patient outcomes were followed up after 12 months to better evaluate pathway accuracy at the time of initial staging. We found both pathways had similar accuracies for identifying patients with metastatic disease and the nature of first major treatment decision was similar. Notably, WB-MRI was more efficient and reduced the number of tests needed, time to complete staging, and costs.

**Implications of all the available evidence**

WB-MRI staging pathways have similar accuracy to current standard staging pathways, resulting in the same treatment decisions. However, they are more efficient and reduce test numbers, time to complete staging, and costs. WB-MRI is, therefore, more suitable for staging in routine clinical practice. Future research should investigate the use of WB-MRI treatment response assessment and cancer surveillance after curative treatments.

Modern MRI scanners can image the entire body within 1 h, and whole-body MRI (WB-MRI)—which typically scans from the head to mid-thigh—is a potentially more accurate and safer alternative to standard multimodality staging pathways. WB-MRI could also accelerate staging, thereby increasing efficiency by reducing additional tests, staging time, and costs. Meta-analyses suggest accuracy for metastatic disease is equivalent to, or might exceed, standard technologies,^7^, ^8^, ^9^, ^10^, ^11^, ^12^, ^13^, ^14^, ^15^, ^16^, ^17^, ^18^ but most combine disparate cancers^7^, ^8^, ^9^, ^11^, ^12^, ^14^, ^15^ or focus on metastasis detection in a single organ,^10^, ^13^, ^16^, ^17^, ^18^ or both. No meta-analysis has considered colorectal cancer staging in isolation; the largest primary study to date included only 20 patients.^19^ Primary studies are predominantly small, single site, explanatory studies with WB-MRI interpretation by a few highly experienced radiologists, which is unlike real-world pathways.^20^ Studies usually compare single modalities (eg, WB-MRI *vs* PET-CT) instead of the multiple staging tests encountered in daily practice.^20^ There are no data regarding how WB-MRI pathways influence staging times, additional tests, costs, or treatment decisions. As such, there is insufficient evidence to assess whether WB-MRI should be adopted.^21^

We did two parallel prospective multicentre trials to elucidate and directly compare the diagnostic accuracy and efficiency of WB-MRI-based staging pathways with standard pathways in non-small-cell lung cancer (Streamline L)^22^ and colorectal cancer (Streamline C). Here, we report findings from Streamline C.

## Methods

### Study design and participants

Streamline C is a multicentre, prospective trial comparing diagnostic accuracy for metastatic disease of staging pathways based on initial WB-MRI, with standard pathways in colorectal cancer. Ethics committee approval was granted on Oct 3, 2012, and the trial was coordinated by Cancer Research UK and University College London Cancer Trials Centre, with oversight from an independent data monitoring committee and a trial steering committee. All patients gave written informed consent.

Patients were recruited from 16 general and teaching UK National Health Service (NHS) hospitals in England. Because eight of the 16 sites did not have the infrastructure to do WB-MRI, these sites sent patients to a nearby hospital for scanning (appendix p 2). Eligible patients were aged 18 years or older with histologically proven or suspected colorectal cancer, referred for staging. Suspicion of colorectal cancer was defined as the presence of a mass on endoscopy or imaging (or both), triggering staging investigations; those without a final diagnosis of cancer were subsequently excluded. Patients were ineligible if they could not provide informed consent, had severe systemic disease making it undesirable to participate, were pregnant, had contraindications to MRI, or had a polyp cancer.

Participants were identified from outpatient clinics, multidisciplinary team meetings, and inpatient wards by the local research team, who took informed consent from consecutive, unselected, eligible patients. A screening log detailed all patients approached and reasons for non-participation, where applicable. Age, performance status, sex, and request date for the first staging investigation were collected from recruited patients. Staging completion date was also recorded, defined as the date of the final test in the standard staging pathway.

The protocol has been published^20^ and is available online.

### Procedures

Participants had contemporaneous WB-MRI plus all standard staging investigations done as part of usual clinical care. Standard investigations were generally undertaken at the recruitment site, or a secondary hospital by referral in the case of specialised tests (such as PET-CT), and were interpreted by local consultant radiologists as per usual clinical practice. Interpretation of standard investigations was masked to WB-MRI images and findings. Although UK NICE guidelines recommend staging chest abdomen and pelvic CT, and pelvis MRI in the case of rectal cancer^4^, case report forms included the nature and date of all standard investigations actually done before the first major treatment decision, and their findings regarding presence and location of any metastatic disease.

The platform used for WB-MRI was in line with usual practice. A minimum dataset of sequences was acquired, including diffusion, T2-weighted, and T1-weighted (pre-intravenous and post-intravenous gadolinium containing contrast medium) imaging (appendix p 3). WB-MRI datasets were uploaded electronically to a secure central imaging server (3Dnet; Biotronics3D, London, UK) for interpretation, and were withheld initially from the local Picture Archiving and Communications System to ensure local radiologists interpreting standard staging investigations were masked.

Across all recruitment sites and imaging hubs, 19 radiologists interpreted WB-MRI and were unaware of all other standard staging investigations and clinical information (other than the suspected cancer diagnosis and its segmental location). All radiologists were fellows of the Royal College of Radiologists and had interpreted at least 20 validated staging WB-MRIs. Radiologists with experience of fewer than 100 WB-MRI datasets initially had their reports validated by more experienced colleagues (ie, those who had worked on >100 WB-MRI datasets) and reported alone only once deemed competent by their colleague. This procedure was designed specifically to mirror how WB-MRI would be reported in NHS practice if more widely disseminated. Radiologists completed case report forms documenting the T and N stage of the local tumour (as per TNM 7th edition^23^), and the presence, location, and diameter of metastatic disease across various anatomical sites using six numerical confidence levels grouped subsequently into normal, equivocal, and abnormal. Radiologists interpreted WB-MRI as per their usual practice, considering known morphology and characteristics of metastatic disease across the various MRI sequences,^24^ and reproduced case report form findings in a free text clinical report, uploaded onto the 3Dnet software for subsequent release to the multidisciplinary team meeting. If additional tests were recommended for equivocal findings, this suggestion was included in their report.

Patients were discussed in the multidisciplinary team meeting at their local hospital as per usual care pathways. WB-MRI images and reports were withheld until patients had completed all standard staging investigations so that the multidisciplinary team made its first major treatment decision based only on standard staging.^20^ The decision was documented (appendix p 4), along with the TNM stage assigned. In the same meeting, the WB-MRI report and images were then shown to the multidisciplinary team via 3Dnet. The team considered the report and images and stated whether additional tests would have been requested before the first major treatment decision could be reached, had WB-MRI been the initial staging investigation (eg, to investigate equivocal findings). Any such tests were then done if they or an equivalent test had not already been done as part of the standard pathway and the multidisciplinary team considered them essential to patient care. If done already, their results were noted. The team recorded the TNM stage based on the WB-MRI staging pathway (ie, WB-MRI plus the results of any additional tests generated, if any) and stated what the first major treatment decision would have been on the basis of this pathway. The final multidisciplinary team treatment decision was then made based on all available tests (ie, standard pathway, WB-MRI, and any additional tests; appendix p 4).

We devised a reference standard using multidisciplinary consensus panel review, a procedure that is standard for diagnostic test accuracy studies where an independent reference standard does not exist or is impossible because of incorporation bias.^20^, ^25^ Patients were followed up for 12 months (or until death, if sooner). Each recruitment site convened a series of panels to derive the reference standard TNM stage, consisting of at least two radiologists (one external to the site) with expertise in cross-sectional imaging and nuclear medicine, and an oncologist or colorectal surgeon, or both. The panel had access to a histopathologist if required, and a member of the Cancer Research UK and University College London Cancer Trials Centre and trial management group attended to ensure the consensus process was uniform across the trial. The panel considered all available clinical data over the follow-up period, including images and results of all staging and follow-up investigations, surgical findings, histopathology (surgical resections and biopsies), and patients' clinical course, and assigned a TNM stage for the time of recruitment. The location and size of any metastatic deposits were recorded. In the absence of histological proof, metastatic disease was assumed if new lesions appeared during follow-up with suggestive imaging characteristics, or if compatible lesions that were already present either progressed or responded to therapy. Specific criteria were applied depending on length of follow up (in the case of death) and if the primary tumour remained in situ (appendix p 5). From all follow-up data, the panel assigned a retrospective optimal primary treatment decision, noting radiological perceptual errors in the initial interpretation of staging investigations (ie, unreported metastases that could be identified by the panel in retrospect, with full knowledge of all follow-up investigations).

### Outcomes

The primary outcome was the difference in per-patient sensitivity for metastatic disease detection between standard and WB-MRI staging pathways, compared against the consensus reference standard. Prespecified outcomes were reported according to the diameter of the largest metastatic deposit (≥1 cm or <1 cm) to assess the effect of lesion size on diagnostic accuracy, per-organ sensitivity, and for WB-MRI as a stand-alone investigation based on the original radiologist report.

Secondary outcomes were difference in per-patient specificity for metastatic disease detection between standard and WB-MRI staging pathways, agreement between treatment decisions based on alternate pathways and the multidisciplinary team and consensus panel treatment decisions, staging efficiency (time taken, test number, and costs), per-organ sensitivity and specificity for metastases, and per-patient agreement for local T and N stage. Additional secondary outcomes related to the effect of differing combinations of MRI sequences on accuracy, interobserver variability in WB-MRI interpretation, and the effect of adding WB-MRI to standard pathways, and will be reported elsewhere. The comparative patient experience of staging pathways and the findings of a discrete choice experiment have already been reported.^26^, ^27^, ^28^

### Statistical analysis

Using methods for comparative studies,^29^ we estimated that 290 patients would give 80% power to detect a clinically meaningful sensitivity difference of 10% between WB-MRI (85%) and standard pathways (75%), assuming 40% metastatic prevalence, 73% concordance between pathways, and 10% withdrawal rate at 1 year, giving a target sample size of 322 patients. The observed withdrawal rate was 19%; therefore, on Dec 7, 2015, as recommended by the independent data monitoring committee, the target sample size was revised to 360 patients to ensure about 290 patients were evaluable.

We report our prespecified primary and secondary outcomes, and additional sensitivity analyses. Binary comparisons (sensitivity, specificity, and treatment decision agreement) were calculated using paired proportions (population marginal) in STATA 14.2 (College Station, TX, USA). For the primary outcome, equivocal disease was considered positive for colon cancer and negative for rectal cancer, as specified by the independent data monitoring committee. Sensitivity analysis treated equivocal results as either negative or positive (additional analysis).

There were no missing data for the primary outcome. Statistical significance was determined on the basis of 95% CIs from Newcombe paired proportion method;^30^ McNemar's test p values are reported. Pathway treatment decisions were grouped for analysis (appendix p 6) and compared to the final treatment decisions made by the multidisciplinary team and consensus panel (as a sensitivity analysis). Extra post-hoc analysis presented the primary tumour site divided into rectum and colon. Time to complete staging pathways (excluding initial diagnostic tests) was calculated in days, by adding times for staging tests (from request to performance) to median wait times for a treatment decision by the multidisciplinary team, calculated across all patients. In the case of missing data, median times from the same or similar tests were used. The median difference in time and number of staging tests between pathways was compared for each patient with 95% CI from 2·5 and 97·5 centiles of 1999 bootstrap samples, with replacement used to compare between standard and WB-MRI staging pathways. Descriptive analysis of time to complete staging are reported in median days with IQR for staging pathways.

We compared the costs of WB-MRI versus standard pathways (appendix p 7). The cost analysis was based on a UK NHS perspective. Costs were calculated in pounds sterling (as of 2016–17) and were inflated as necessary. The time horizon was the time from initial diagnosis to treatment decision by the multidisciplinary team. Given the time horizon, which was less than 1 year, discounting was not applied. We calculated the mean cost per patient of tests received when undergoing standard imaging pathways only and WB-MRI (including additional staging tests ordered after the WB-MRI). We only included the cost of the tests received; the costs of the multidisciplinary team were not included because this cost was incurred irrespective of the type of staging test received. We did not include any adverse events related to imaging because no such events were reported. Unit costs were taken from 2016–17 NHS reference costs.^31^ Decisions about which reference costs to use were made with appropriate clinical input (appendix p 8). Mean per-patient staging costs for standard pathways and WB-MRI were compared using 95% CIs derived from 1000 bootstrapped replications of the mean with replacement

Streamline C is registered with the International Standard Randomised Controlled Trial registry, number ISRCTN43958015.

### Role of the funding source

The funder of the study stipulated that the study design should be a diagnostic accuracy trial using a cohort design, but was not involved in data collection, data analysis, data interpretation, or writing of the report. The corresponding author had full access to all the data in the study and had final responsibility for the decision to submit for publication.

## Results

**Figure 1:**
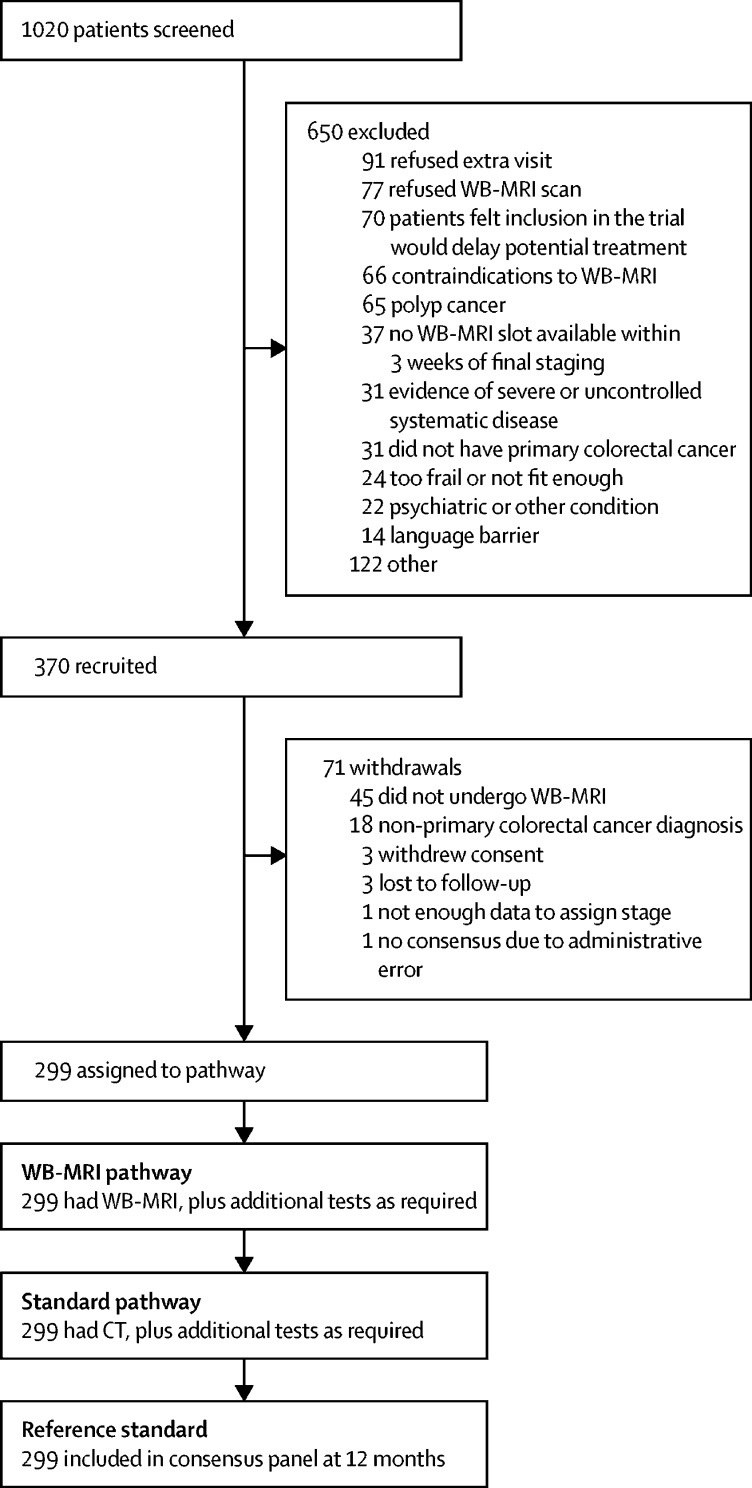
Trial profile WB-MRI=whole-body MRI.

Between March 26, 2013, and Aug 19, 2016, 1020 patients were screened for eligibility (figure 1). 370 patients were recruited, of which 71 were excluded. The final cohort of 299 patients had a median age of 65 years (IQR 57–71) and 106 (35%) were women (figure 1, table 1). According to the consensus reference standard, 288 (96%) patients were stage T2 or above, 166 (56%) were node-positive (appendix p 9), and 68 (23%) had metastatic disease at the time of staging (48 [71%] of 68 had liver metastasis; appendix p 10). In six patients with metastatic disease at the time of staging (according to protocol definitions; appendix p 5), metastasis only became apparent during follow-up and was not visible on initial staging investigations, even in retrospect.

**Table 1:** Baseline characteristics of final trial cohort

		**Value**
**Sex**		
Male		193 (65%)
Female		106 (35%)
**Age, years**		
Median (IQR)		65 (57–71)
Range		30–90
**Performance status**		
Fully active		199 (67%)
Ambulatory		
	Able to work	31 (10%)
	Not able to work	3 (1%)
Not recorded		66 (22%)
Tumour location*		
Rectum		130 (43%)
Sigmoid		86 (29%)
Descending		11 (4%)
Transverse†		24 (8%)
Ascending		29 (10%)
Caecum		43 (14%)

Data are n (%) unless otherwise stated.

* By consensus reference standard. Some patients have multiple tumour locations.

† Flexure tumours were combined and categorised as transverse colon.

**Figure 2:**
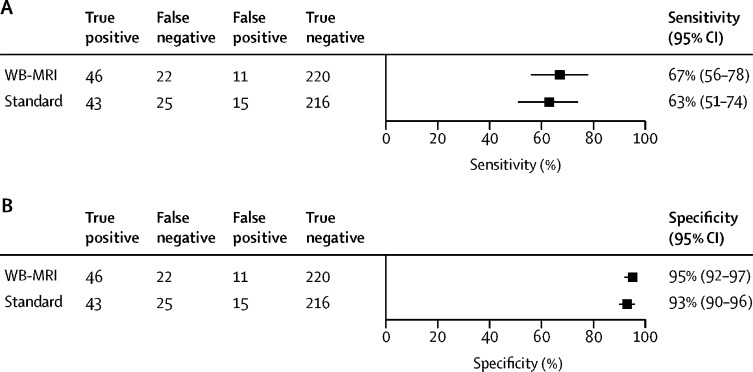
WB-MRI and standard staging pathways sensitivity and specificity for patients with metastatic disease against the consensus reference standard WB-MRI=whole-body MRI.

Sensitivity of staging for patients with metastatic disease was 67% (95% CI 56–78) for WB-MRI and 63% (51–74) for standard pathways, a difference of 4% (−5 to 13, p=0·51; table 2, figure 2). For the primary outcome, there were three perceptual errors in the WB-MRI pathway and six in the standard pathway. No adverse events (serious or non-serious) were reported during the trial.

**Table 2:** Per-patient sensitivity and specificity for metastatic disease

	Patients with metastatic disease*	**Sensitivity**				Patients without metastatic disease*	**Specificity**			
		WB-MRI staging pathway†	Standard staging pathway	Difference	p value		WB-MRI staging pathway†	Standard staging pathway	Difference	p value
Diagnostic accuracy‡	68	67% (56 to 78)	63% (51 to 74)	4% (−5 to 13)	0·51	231	95% (92 to 97)	93% (90 to 96)	2% (−2 to 6)	0·48
Equivocal lesions considered positive	68	71% (59 to 80)	68% (56 to 78)	3% (−6 to 12)	..	231	95% (91 to 97)	92% (88 to 95)	3% (−2 to 7)	..
Equivocal lesions considered negative	68	65% (53 to 75)	58% (46 to 68)	7% (−2 to 17)	..	231	98% (94 to 99)	98% (95 to 99)	0% (−3 to 2)	..

Data are n or % (95% CI).

* Patients by consensus reference standard.

† WB-MRI plus additional generated tests.

‡ Equivocal results considered positive for colonic tumours and negative for rectal tumours.

Specificity did not differ between the WB-MRI pathway (95% [95% CI 92–97]) and standard pathway (93% [90–96], p=0·48). Sensitivity analysis found no significant differences between pathways when lesions reported as equivocal were treated as either all positive or all negative (table 2), or across individual organ sites (appendix p 11). The WB-MRI pathway had 86% (95% CI 74–94) sensitivity for patients whose largest metastasis was at least 1 cm, which did not differ from standard pathways (82% [69–91]; appendix p 12). As a stand-alone investigation (ie, without additional tests generated) WB-MRI had a similar sensitivity to that of the standard pathway, but had lower specificity than the standard pathway (appendix p 13).

The WB-MRI pathway had 54% agreement for T stage compared with 60% for the standard pathway, a non-significant difference of 6% (95% CI 0–12; appendix p 14). N stage agreement did not significantly differ between the pathways (appendix p 15). Agreement with the final treatment decision of the multidisciplinary team was 96% for WB-MRI and 95% for the standard pathway (table 3). Treatment decisions based on WB-MRI and standard pathways had similar levels of agreement with the retrospective consensus panel optimal treatment decision for rectal and non-rectal cancers (appendix p 16).

**Table 3:** Agreement between pathway and multidisciplinary team treatment decisions

	n*	**WB-MRI staging pathway**†		**Standard staging pathway**		**Difference agreement, % (95% CI)**
		Agreement	Disagreement	Agreement	Disagreement	
**Colorectal cancer**						
All patients	296	284 (96%)	12 (4%)	282 (95%)	14 (5%)	1% (−2 to 4)
**Colon cancer**						
All patients	168	166 (99%)	2 (1%)	165 (98%)	3 (2%)	1% (−3 to 4)
Patients with metastatic disease	33	33 (100%)	0 (0%)	32 (97%)	1 (3%)	3% (−6 to 12)
Patients without metastatic disease	135	133 (99%)	2 (1%)	133 (99%)	2 (1%)	0% (−4 to 4)
**Rectal cancer**						
All patients	128	118 (92%)	10 (8%)	117 (91%)	11 (9%)	1% (−5 to 7)
Patients with metastatic disease	32	28 (88%)	4 (12%)	28 (88%)	4 (12%)	0% (−10 to 10)
Patients without metastatic disease	96	90 (94%)	6 (6%)	89 (93%)	7 (7%)	1% (−7 to 9)

Data are n (%) unless otherwise stated.

* Three patients were missing at least one type of patient treatment decision.

† WB-MRI plus additional generated tests.

Across the cohort, standard staging pathways involved 558 individual investigations and WB-MRI involved 320 individual investigations; WB-MRI pathways generated an additional 21 tests (appendix pp 17–18). WB-MRI pathways required fewer tests (median, one [95% CI 1 to 1]) than did standard pathways (two [2 to 2]), a difference of one (1 to 1; appendix p 19).

**Figure 3:**
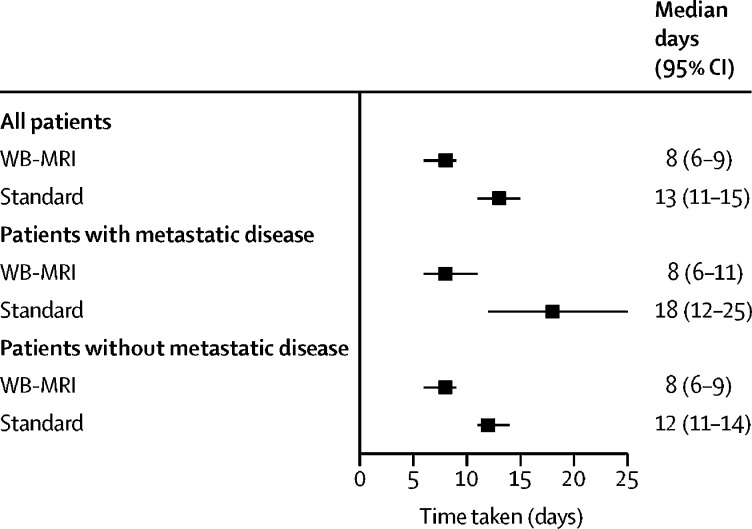
Time taken for staging pathways WB-MRI=whole-body MRI.

Time to staging was shorter for WB-MRI pathways than for standard pathways (median, 8 days [IQR 6–9] *vs* 13 days [11–15]); a difference of 5 days (3–7; figure 3, appendix pp 20–21). Mean per-patient costs for the WB-MRI pathway (£216 [95% CI 211–221]) were lower than for the standard staging pathway (£285 [260–310]; appendix p 22).

## Discussion

To date, Streamline C is the largest prospective, multicentre trial to compare the diagnostic accuracy of WB-MRI and standard staging pathways for metastatic disease in patients newly diagnosed with colorectal cancer. Both pathways showed similar accuracy, but the WB-MRI pathway was more time-efficient and cost-efficient. Treatment decisions were similar. Our data suggest WB-MRI is a viable and desirable replacement for standard pathways.

WB-MRI pathways had no advantage over standard pathways in terms of diagnostic accuracy. The overall sensitivity of WB-MRI pathways (and WB-MRI alone) for metastatic disease was lower than published meta-analyses suggest. For example, Xu and colleagues^7^ reported per-patient sensitivity and specificity of WB-MRI as 86% (95% CI 70–94) and 97% (94–99), respectively, in a meta-analysis of nine studies. However, primary studies considered a wide range of primary cancers, were single-site explanatory studies, and all except one recruited fewer than 150 patients. The largest previous study^19^ of WB-MRI for colorectal cancer staging recruited just 20 patients. Our consensus reference standard considered a follow-up of 12 months and several patients showed metastases during this period that were not visible, even in retrospect, on any imaging modality. Such disease is currently beyond the resolution of cross-sectional imaging and, in part, explains the large number of patients who relapse after attempted curative resection. The number of perceptual errors was low, and many retrospectively visible lesions were subtle and difficult to detect prospectively. As a pragmatic trial, Streamline C provides the best estimate of colorectal cancer staging accuracy in routine clinical practice.

We found that the WB-MRI pathway had 86% sensitivity for patients with metastatic disease of at least 1 cm. 48 (71%) of 68 patients with metastasis had liver metastasis, and the association between diameter and detection is well established. In their 2010 meta-analysis, Niekel and colleagues^32^ reported that sensitivity of even dedicated liver MRI is often below 50% for liver metastases that are less than 1 cm. It is important to differentiate WB-MRI staging protocols from those intended specifically to stage the liver. Our WB-MRI protocol complied with accepted international standards, including diffusion weighted imaging and post-gadolinium sequences; however, by necessity, we had to compromise—for example, on slice thickness—to ensure reasonable total scan times. Although the addition of liver-specific contrast agents to standard protocols is feasible and will probably improve sensitivity, costs might be prohibitive in some health-care settings.

We found that WB-MRI pathways had similar accuracy for T and N staging compared with standard pathways. Although MRI appears to be promising for local staging of colorectal cancer, its superiority over CT is unproven.^33^ Agreement with both the final multidisciplinary team treatment decision and the optimal retrospective treatment decision was similar for both staging pathways, suggesting that WB-MRI could replace standard pathways without patient detriment.

Generally, efficiency receives less attention than diagnostic accuracy.^21^ Streamline C found that WB-MRI pathways were more efficient than standard pathways, substantially reducing the number of tests needed and time to complete staging. These changes affected costs, with average per-patient staging costs decreasing by £69. Although it is unlikely that shortening staging time by a few days will directly affect patient outcomes, prolonged pathways increase anxiety so any reduction is advantageous.^6^ Although access to MRI is restricted in many health-care settings, our data suggest that increased provision would ultimately reduce the cost and complexity of staging colorectal cancer. A discrete choice experiment done as part of the trial shows patients generally prefer WB-MRI staging to standard pathways, if they reduce staging test number, staging times, and radiation exposure as found in Streamline C.^28^

A strength of our trial is its pragmatic design. We recruited from a representative range of general and teaching hospitals, with all imaging done and interpreted according to usual local protocols, to increase generalisability of our results. The 19 radiologists interpreting WB-MRI were representative of those who would do so in daily NHS practice. We avoided using a smaller number of highly experienced radiologists; although we acknowledge that such individuals might achieve sensitivities greater than we report, they do not represent the national workforce. We used multidisciplinary team meetings to mirror patient care in the NHS. In doing so, we captured the entirety of standard pathways, including contemporaneous treatment decisions. We used a novel cloud-based image repository to maintain blinding and control multidisciplinary team access to WB-MRI until the appropriate time in the decision-making process. We were able to model the content and timing of WB-MRI staging pathways, and the potential effect on decision making. Conversely, previous research usually reports head-to-head comparisons between single imaging platforms, failing to capture pathway complexity. To our knowledge, our trial design is unique.

Streamline C does have limitations. We masked radiologists to patient history and, for WB-MRI, to contemporaneous imaging, which was necessary to isolate diagnostic test accuracy within a pragmatic setting. Participants were representative of those undergoing staging in daily practice, although we did exclude pregnant women, patients not wanting to undergo WB-MRI, and patients with contraindications to MRI. The prevalence of metastatic disease was lower than assumed by our power calculation. However, the independent data monitoring committee recommended continuing the trial so as to achieve our original target number of evaluable patients. On independent data monitoring committee advice, equivocal findings were treated as negative for rectal cancer (as many undergo chemoradiation, allowing such lesions to be characterised over time). Sensitivity analysis found that alternate classification of equivocal abnormalities had no meaningful effect. We modelled timing of WB-MRI staging pathways on the basis of real waiting times collated from recruitment sites during the trial, although sites had capacity to do WB-MRI. Waiting times might not be representative of those at other hospitals, and in other countries. Some of the benefits of reduced staging time by WB-MRI pathways could be negated if time to commencing treatment (eg, surgical resection) are not reduced in parallel. Treatment decisions based on WB-MRI pathways were made after the multidisciplinary team was unmasked to all standard imaging tests, which could introduce bias. However, this situation was unavoidable if the full complexity of standard staging pathways was to be captured without interference from WB-MRI findings and if treatment decisions were to be recorded contemporaneously. Furthermore, alternate pathway agreement with a retrospective optimal treatment at 12 months remained very similar. Our cost analyses reflect an English NHS perspective and could differ in other settings, which might negate some of the cost advantages of WB-MRI pathways. Although WB-MRI is advocated as being safer than current standard staging investigations, new technologies are reducing radiation dose,^34^ and there are current uncertainties about the neuronal deposition of gadolinium.^35^ Further research is needed to define the potential use of WB-MRI in the assessment of treatment response and post-therapy surveillance for recurrent disease. Our findings are specific to colorectal cancer and might not be relevant to other primary tumour sites.

In summary, WB-MRI staging pathways have similar diagnostic accuracy to standard pathways for identifying patients with metastatic disease in newly diagnosed colorectal cancer, and precipitate similar treatment decisions. However, they reduce staging time, test number, and costs. In a real-world NHS setting, WB-MRI-based pathways are a viable replacement for standard pathways.

## Data sharing

Individual participant data that underlie the results reported in this Article, after de-identification (text, tables, figures, and appendices), will be available for individual participant data meta-analysis beginning 9 months and ending 36 months after article publication. Data will be available to investigators whose proposed use of the data has been approved by an independent review committee (learned intermediary) identified for this purpose. Data access requires proof of relevant ethical committee approval for the specified analysis only. Data will be limited to those required for a specific analysis to protect deanonymisation. Where proposals that would compete with ongoing or planned research from the investigators within the trials team, data access will only be granted once investigator team publications are submitted. Proposals should be directed to the corresponding author; to gain access, data requestors will need to sign a data access agreement. After 36 months, there is no funded technical support. Information regarding submitting proposals and accessing data can be obtained by emailingctc.enquiries@ucl.ac.uk
