# Supplementary material for: Diagnostic accuracy of whole-body MRI versus standard imaging pathways for metastatic disease in newly diagnosed colorectal cancer: the prospective Streamline C trial
Source: Lancet Gastroenterol Hepatol. 2019 May 9;4(7):529–37. doi: 10.1016/S2468-1253(19)30056-1 (PMC6547166; doi:10.1016/S2468-1253(19)30056-1)
Supplement: Supplementary appendix [file mmc1.pdf]

THE LANCET
Gastroenterology & Hepatology

Supplementary appendix

This appendix formed part of the original submission and has been peer reviewed. We post it as supplied by the authors.

Supplement to: Taylor SA, Mallett S, Beare S, et al. Diagnostic accuracy of whole-body MRI versus standard imaging pathways for metastatic disease in newly diagnosed colorectal cancer: the prospective Streamline C trial. *Lancet Gastroenterol Hepatol* 2019; published online May 9. http://dx.doi.org/10.1016/S2468-1253(19)30056-1.

Online appendix

Contents

Streamline Investigators 1
Recruitment sites and Imaging hubs 2
WB-MRI protocol minimum dataset 3
Summary of information recorded by the MDT and categories of treatment decisions 4
Criteria for diagnosis of metastatic disease by the consensus reference panel 5
Grouping of treatment decision for analysis 6
Justification for economic analysis 7
Streamline C Test Unit costs from NHS Reference Costs 2016/17 8
Appendix Table 1: T and N stage of the final trial cohort based on consensus reference standard 9
Appendix Table 2: Organ sites of metastatic disease according to the consensus reference standard 10
Appendix Table 3: Per organ sensitivity and specificity for metastatic disease- WB-MRI staging pathways versus standard staging pathways against the consensus reference standard 11
Appendix Table 4: Per patient sensitivity and specificity for metastatic disease according to the size of the largest deposit- WB-MRI staging pathways versus standard staging pathways, against the consensus reference standard 12
Appendix Table 5: Per-patient sensitivity and specificity for metastatic disease-WB-MRI as a standalone investigation versus standard staging pathway, against the consensus reference standard 13
Appendix Table 6: Per-patient agreement for tumour T stage- WB-MRI staging pathways versus standard staging pathways against the consensus reference standard 14
Appendix Table 7: Per-patient agreement for tumour N stage- WB-MRI staging pathways versus standard staging pathways against the consensus reference standard 15
Appendix table 8: Agreement between treatment decisions based on the WB-MRI and standard staging pathways and the retrospective 12-month consensus panel optimal treatment decision 16
Appendix Table 9: Investigations performed as part of the standard staging pathway 17
Appendix Table 10: Additional Investigations generated by WB-MRI 18
Appendix Table 11: Number of tests required to complete staging according to the staging pathway 19
Appendix Table 12: Time to complete staging according to staging pathway (95% CI) 20
Appendix Table 13: Time to complete staging according to staging pathway-interquartile range 21
Appendix Table 14: Mean per patient staging cost according to staging pathway 22

Streamline Investigators

Ruth Evans, Simon Ball, Revanth Jannapureddy, Tina Mills-Baldock, Kishor Barhate, Zoltan Nagy, Sherif Raouf, Akosa Aboagye, Girija Anand, Rommel Butawan, Elizabeth Hadley, Adesewa Onajobi, Kathryn Tarver, Tanjil Nawaz, Catherine Norman, Nathalie Rich, Sidra Tulmuntaha, Shafi Ahmed, Louise Lim, Fiona McKirdy, Jenna Couture, Shahanara Ferdous, Payal Julka, Ali Mohammed, Terry O'Shaughnessy, William Ricketts, Ruth Evans, Marie Jackson, Clive Kay, Andy Lowe, Janet McGowan, Amjad Mohammed, Jon Robinson, Lara Curry, Sasithar Maheswaran, Subramanian Ramesh, Pippa Riddle, Shaki Balogun, Yvonne Campbell, Nelesh Jeyadevan, Aji Kavidasan, Imogen Locke, Tuck-Kay Loke, Ibiyemi Olaleye, Clare Collins, Elizabeth Green, Colm Prendergast, Thida Win, Amy Davis, Lyn Blakeway, Sofia Gourtsoyianni, Adrian Green, Christian Kelly-Morland, Sahar Naaseri, Davide Prezzi, David Snell, Dorothee Boisfer, Keyury Desai, Balinder Hans, Sophia Hans, Eleni Ntala, Adnam Alam, Stephen Burke, Angshu Bhowmik, Nishat Bharwani, Gule Hanid, Lesley Honeyfield, Tina Stoycheva, Nicola Strickland, Farid Bazari, Helen Beedham, Jane De Los Reyes Lauigan, Priya Limbu, Nicola Lucas, Sally O'Connor, Anita Rhodes, Laletha Agoramoorthy, Martha Handousa, Abel Jalloh, Stefania Stegner, Shanna Wilson, David Birch, Suzanne Chukundah, Priscilla Phiri, Raj Srirajaskanthan, Eleni Karapanagiotou, Daniel Smith, Ferrial Syeed, Chloe van Someren, Rudi Borgstein, Jamila Roehrig, David Chao, Lorraine Hurl, Andrew Gogbashian, Andre Nunes, Ian Simcock, James Stirling, Richard Beable, Maureen Furneaux, Nicola Gibbons, Antony Higginson, Howard Curtis, Kitrick Perry, Anita Amadi, Heather Hughes, Prital Patel, Gary Atkin, Colin Elton, Stephen Karp, Lisa Woodrow, Dominic Yu, Sajid Khan, Alistair Rienhardt, Pooja Datt, Rajapandian Ilangovan, Ian Jenkins, Saba Mahmud, Teresa Light, Joanne Kellaway, Ann O'Callaghan, William Partridge, Amelia Daniel, Ugo Ekeowa, Michael Long, Peter Russell, Erica Scurr, Veronica Morgan, Nina Tunariu, Elizabeth Chang, Laura Hughes, Ellice Marwood, Katie Prior, Meena Reddi, Kara Sargus, Abby Sharp, Teresita Beeston, Elizabeth Isaac, Adoracion Jayme, Jagadish Kalasthry, Wivijin Piga, Farzana Rahman, Shraddha Weir, Aileen Austria, James Crosbie, Alec Engledow, Jonathan McCullogh, Austen Obichere, Kai-Keen Shiu, Christopher Wanstall, Celia Simeon, Amy Smith, Andrew Bateman, David Breen, Liane Davis, Chris Everitt, Alice Johnson, Paul Nichols, Beth Shepherd, Kayleigh Gilbert, Azmina Verjee, Michelle Saull, Jonathan Wilson, Rashidat Adeniba, Veronica Conteh, Sarah Howling, Sara Lock

Recruitment sites and Imaging hubs

Recruitment site	Imaging hub
University College Hospital London	University College Hospital London
Barnet Chase Farm Hospitals[a]	
Princess Alexandra Hospital, Harlow	
North Middlesex	
St Marks Hospital, Harrow	
West Middlesex Hospital	
Whittington Hospital	
Queen's Hospital. Romford	
Homerton Hospital[b]	Homerton Hospital
St Bartholomews Hospital/Royal London Hospital	St Bartholomews Hospital/Royal London Hospital
Charing cross Hospital	Charing cross Hospital
Southampton Hospital	Southampton Hospital
Queen Alexandra Hospital Portsmouth	Queen Alexandra Hospital Portsmouth
Guy's and St Thomas Hospital	Guy's and St Thomas Hospital
Lewisham Hospital	
Bradford Royal Infirmary	Bradford Royal Infirmary

[a]1 patient underwent WB-MRI at the Paul Strickland Scanner Centre, Mount Vernon

[b] 10 patients underwent WB-MRI at St Bartholomew's Hospital

WB-MRI protocol minimum dataset
Scanning maybe performed at either 1.5T or 3T.
Whole body is head to mid-thigh

1. Whole-body diffusion weighted imaging:

Axial: STIR-EPI (or other fat sat technique) diffusion weighted imaging. Fixed slice thickness of 5mm to 7mm (to match T2 and T1 weighted axials as below) two b-values (b50 and b900). A minimum acquisition matrix of 128 x 128 (or an interpolated equivalent) (rectangular FOV should be used if available and appropriate for the patient), as a reference a minimum SNR of 6 on b50 images (for liver) should be maintained by increasing the number of averages. All imaging should be performed in gentle respiration (recommended as 4 stations of 50 slices beginning from the vertex to mid thighs). Diffusion imaging through the brain is optional.

2. Whole-body T2 weighted imaging:

Axial*:* Axial T2 weighted (without fat-suppression) imaging, maximum 5 to 7 mm slice thickness. Where possible, respiratory and ECG triggering should be used for the chest, respiratory triggering alone for the upper abdomen.

Pre-contrast T1 weighted imaging:

DIXON Technique to be applied if available.

a. **Axial**: Whole-body T1 GRE (e.g. Flash 2D) non-contrast enhanced non fat sat. Image resolution and slice thickness should be ideally matched to T2 weighted imaging.

OR

b. **Coronal**: T1 fat saturated volume interpolated gradient echo imaging (e.g. 3D) pre contrast.

Post-contrast T1 weighted imaging (if gadolinium not contraindicated or refused):

Minimum data set
Axial liver (60-70 sec)
Axial lung (equilibrium phase)
SFOV axial head
Optional
Coronal (organ specific or whole body)

a. **Axial:** post contrast e.g. T1 fat saturated volume interpolated gradient echo imaging (3D) breath hold of the *liver (60-70 seconds delay) and lungs*. Multiple breath-holds employed to provide full volume coverage if required. A minimum of a 256x256 (rectangular FOV acquisition if possible and appropriate for the patient) acquisition matrix should be employed. 5-7 mm slice thickness.

b. **Coronal:** post contrast whole body; e.g. T1 fat saturated volume interpolated gradient echo imaging (3D) and post contrast. Slice thickness 5mm. Breath Hold.

c. **Axial:** fat saturated T1 weighted imaging of the brain (SFOV). An acquisition matrix of 256 x 256 should be employed

Summary of information recorded by the MDT and categories of treatment decisions

- Stage and treatment decision based on standard investigations (and the number, timing, nature and findings of these investigations).
- Stage and theoretical treatment decision based on WB-MRI staging pathway (and the number, timing, nature and findings of additional tests generated, if any).
- Final treatment decision incorporating all available tests.

Categories of treatment decisions

- Surgical removal of primary alone
- Surgery for primary followed by planned adjuvant chemotherapy
- Surgery for primary followed by planned chemotherapy followed by surgical removal of metastasis
- Surgical removal of primary and metastatic site(s) alone
- Surgery for primary and metastatic site(s) followed by planned adjuvant chemotherapy
- Neo-adjuvant chemo (radio) therapy alone
- Neo-adjuvant chemo (radio) therapy alone followed by planned surgical removal of primary
- Neo-adjuvant chemo (radio) therapy alone followed by planned surgical removal of primary and metastatic site(s)
- Palliative care
- Other– Describe

Criteria for diagnosis of metastatic disease by the consensus reference panel

- For patients in whom the primary tumour was completely removed within 3 months of diagnosis, all new metastatic sites identified over the follow up period were assumed to have been present at diagnosis
- If the primary tumour was left in situ for more than 3 months of diagnosis (or there was incomplete removal), new metastatic sites were assumed to have been present at diagnosis if they were identified within 6 months of diagnosis If new metastatic sites were diagnosed beyond 6 months of diagnosis, and there was no evidence of their presence on retrospective review of all staging investigations, they were assumed to be new disease and not present at diagnosis
- If patients with tumours left in situ did not undergo any imaging capable of detecting metastatic disease within 6 months of diagnosis of the primary and new metastatic sites were apparent beyond 6 months but not visible in retrospect on any trial imaging, the consensus panel decided if the disease was likely present at diagnosis, based on its location, size and imaging characteristics.
- If a patient died before the 12 months' follow-up, the panel reviewed all available imaging, histopathology and clinical course prior to death and in consensus decided if a confident diagnosis of the presence or absence of metastatic disease could be made (for example the presence of imaging characteristics compatible with metastasis and no alternative explanation, or if lesions with characteristics compatible with metastasis that either grew or shrunk (on therapy). If this judgement could not be made with confidence (for example if the patient had equivocal lesions on staging investigations and no further follow up), patients were not be excluded but multiple imputation used to account for missing data

Grouping of treatment decision for analysis

**Treatment decision category**	**Treatment decisions included**
Surgery for the primary but no chemotherapy	• Surgical removal of primary alone
Surgery for the primary & chemotherapy (and/or radiotherapy)	• Surgery for primary followed by *planned* Adjuvant chemotherapy<br>• Neo-adjuvant chemo (radio) therapy alone followed by *anticipated* surgical removal of primary
Chemotherapy (and/or radiotherapy) without surgery	• Neo-adjuvant chemo (radio) therapy alone<br>• Palliative care
Surgical metastectomy with or without chemotherapy	• Surgery for primary followed by *planned* chemotherapy followed by surgical removal of mets<br>• Surgical removal of primary and metastatic site(s) alone<br>• Surgery for primary and metastatic site (s) followed by *anticipated* Adjuvant chemotherapy<br>• Neo-adjuvant chemo (radio) therapy alone followed by *anticipated* surgical removal of primary AND metastatic site(s)

Justification for economic analysis

As per the trial protocol, a full economic evaluation was not performed because of the observed concordance between WB-MRI and conventional staging tests in informing treatment decisions. The care pathway may be divided into two stages: the treatment decision pathway and the subsequent disease pathway. The former includes the time from initial diagnosis to treatment decision by the MDT; the latter includes the time period following the treatment decision. If there is no difference in treatment decisions made with the two different staging methods, the only difference in costs assigned to the two staging methods is the differential costs of the two sets of staging tests (i.e. standard staging vs. WB-MRI pathway, including additional tests requested), and there will be no difference in treatment pathways or outcomes on the basis of the experimental staging tests used and the resulting costs. As specified in the trial protocol, concordance between conventional staging and WB-MRI was defined as the situation in which >90% of treatment decisions were the same using both imaging methods, or <10% treatment decision were different. Discordance was defined as the case in which >10% treatment decisions were different, or <90% were the same. In the protocol we specified that in the case of concordance, the economic analysis would focus on the cost of the treatment decision pathways only, because the disease pathways will be no different. In this case the cost-effectiveness of WB-MRI versus conventional staging algorithms depends only on the incremental cost (positive or negative) of WB-MRI versus conventional staging algorithms in the treatment decision pathway. Conversely, if there is discordance between the treatment decisions, suggesting that patients would have received different treatment depending on which of the two experimental staging methods was used, then the economic analysis ought to include both the treatment decision pathways and the subsequent disease pathways because both of these will vary between WB-MRI and conventional staging algorithms. In this case the cost-effectiveness of WB-MRI depends on the incremental cost of the WB-MRI versus conventional staging algorithms in the treatment decision pathway plus the incremental costs and health benefits of the disease pathway.
The agreement with the MDT final treatment decision was 96% and 95% for WB-MRI and standard pathways respectively, clearly indicating there was concordance between conventional staging and WB-MRI. On this basis, as specified in our trial protocol, the economic analysis focused on a comparison of the costs of the treatment decision pathways only, which is the analysis included in the manuscript.

Streamline C Test Unit costs from NHS Reference Costs 2016/17

Currency code	Currency Description	Tests	Mean unit cost (£)
RD03Z	Magnetic Resonance Imaging Scan of One Area, with Pre- and Post-Contrast	MRI liver<br>MRI spine<br>MRI brain	180.35
RD01A	Magnetic Resonance Imaging Scan of One Area, without Contrast, 19 years and over	MRI pelvis<br>MRI pelvis/rectum	139.30
RD05Z	Magnetic Resonance Imaging Scan of Two or Three Areas, with Contrast	MRI - WB-MRI<br>MRI - abdomen and pelvis	206.51
RN03A	Positron Emission Tomography with Computed Tomography (PET-CT) of more than Three Areas, 19 years and over	PET CT	484.17
RD21A	Computerised Tomography Scan of One Area, with Post-Contrast Only, 19 years and over	CT chest	97.39
RD26Z	Computerised Tomography Scan of Three Areas, with Contrast	CT colonography	122.51
YD03Z	Percutaneous Biopsy of Lesion of, Lung or Mediastinum	Surgical biopsy	791.50
RD21A	Computerised Tomography Scan of One Area, with Post-Contrast Only, 19 years and over	CT liver	97.39
RD24Z	Computerised Tomography Scan of Two Areas, with Contrast	CT abdomen and pelvis	112.33
RD26Z	Computerised Tomography Scan of Three Areas, with Contrast	CT chest, abdomen and pelvis<br>CT other	122.51
RN15A	Nuclear Bone Scan of Two or Three Phases, 19 years and over	Bone scan	292.40
RD40Z	Ultrasound Scan with duration of less than 20 minutes, without Contrast	Ultrasound	51.78
YJ04Z	Core Needle Biopsy of Axillary Lymph Nodes	Ultrasound guided biopsy	92.43
RD97Z	Admission or Attendance for Diagnostic Imaging	X ray	18.71
FE35Z	Diagnostic Flexible Sigmoidoscopy, 19 years and over	Sigmoidoscopy	169.43
RD42Z	Ultrasound Scan with duration of 20 minutes and over, without Contrast	Rectal ultrasound	64.95

Appendix Table 1

T and N stage of the final trial cohort based on consensus reference standard

**T Stage**[a]	**N stage**[a]		
	N0	N1	N2
T1	11	0	0
T2	36	12	4
T3	76	62	39
T4	10	17	32

[a]232 patients with histological proof for T and N stage, 6 patients with histological proof for T stage only and 1 patient for N stage only

Appendix Table 2
Organ sites of metastatic disease according to the consensus reference standard

			Imaging diagnosis if no histological proof[b]		
**Organ Site**	**Number of patients[a]**	**Histological proof (n, %)**	**Characteristic imaging appearances (n, %)**	**Growth on follow- up (n, %)**	**Response to therapy (n, %)**
Liver	48	12 (25)	33 (92)	26 (72)	19 (53)
Lung	20	0 (0)	19 (95)	13 (65)	11 (55)
Bone	3	0 (0)	3 (100)	2 (67)	1 (33)
Mesentery/peritoneum	7	2 (29)	4 (80)	4 (80)	1 (20)
Nodal (metastatic)	11	2 (28)	8 (89)	7 (78)	3 (33)
Other [c]	2	1 (33)	2 (100)	2 (100)	0 (0)

[a] 68 patients had metastasis. Patients may have more than 1 site of metastatic disease
[b] Metastasis may fulfil more than 1 criterion
[c]One patient had two additional sites of metastatic disease

Appendix Table 3
Per organ sensitivity and specificity for metastatic disease- WB-MRI staging pathways versus standard staging pathways against the consensus reference standard

	Sensitivity % (CI 95%)[a]				Specificity % (CI 95%)[a]			
**Site**	**Number with metastatic disease[b]**	**WB-MRI staging pathway[c]**	**Standard staging pathway**	**Difference: WB-MRI[c]-Standard**	**Number without metastatic disease[b]**	**WB-MRI staging pathway[c]**	**Standard staging pathway**	**Difference: WB-MRI[c]-Standard**
Liver	48	74 (60 to 84)	72 (57 to 83)	2 (-10 to 14)	251	99 (97 to 100)	99 (97 to 100)	0 (-2 to 2)
Lung	20	55 (34 to 74)	65 (43 to 82)	-10 (-35 to 15)	279	99 (96 to 99)	99 (96 to 99)	0 (-2 to 2)
Bone	3	33 (6 to 79)	0 (0 to 56)	33 (-53 to 120)	296	99 (98 to 100)	99 (98 to 100)	0 (-2 to 1)
Mesentery / peritoneum	7	14 (3 to 51)	14 (3 to 51)	0 (-34 to 34)	292	99 (97 to 100)	99 (97 to 100)	0 (-2 to 2)
Nodal (metastatic)	11	45 (21 to 72)	27 (10 to 57)	18 (-13 to 49)	288	97 (94 to 98)	98 (95 to 99)	-1 (-3 to 1)
Other	2	0 (0 to 66)	0 (0 to 66)	0 (-50 to 50)	297	98 (95 to 99)	98 (96 to 99)	0 (-3 to 2)

[a] equivocal results considered positive for colonic tumours and negative for rectal tumours
[b] Patients by consensus reference standard
[c] WB-MRI plus additional generated tests

Appendix Table 4
Per patient sensitivity and specificity for metastatic disease according to the size of the largest deposit- WB-MRI staging pathways versus standard staging pathways, against the consensus reference standard

<table>
<tr><th></th><th></th><th colspan="3">Sensitivity % (CI 95%)</th><th></th><th colspan="3">Specificity % (CI 95%)</th></tr>
<tr><th></th><th>Number with metastatic disease[a,b]</th><th>WB-MRI staging pathway[c]</th><th>Standard staging pathway</th><th>Difference (P value)</th><th>Number without metastatic disease[a,b]</th><th>WB-MRI staging pathway[c]</th><th>Standard staging pathway</th><th>Difference (P value)</th></tr>
<tr><td>Maximum metastatic deposit size ≥1cm</td><td>45</td><td>86 (74 to 94)</td><td>82 (69 to 91)</td><td>4 (-8 to 17) p=0·69</td><td rowspan="2">231</td><td rowspan="2">95 (92 to 97)</td><td rowspan="2">93 (90 to 96)</td><td rowspan="2">2 (-2 to 6) p=0·48</td></tr>
<tr><td>Maximum metastatic deposit size <1cm</td><td>20</td><td>35 (18 to 57)</td><td>30 (15 to 52)</td><td>5 (-13 to 23) p>0.99</td></tr>
</table>

[a] Patients by consensus reference standard
[b]3 patients missing largest metastatic disease size
[c]WB-MRI plus additional generated tests

Appendix Table 5

Per-patient sensitivity and specificity for metastatic disease- WB-MRI as a standalone investigation versus standard staging pathway, against the consensus reference standard

	Sensitivity % (CI 95%)[a]				Specificity % (CI 95%)[a]			
	Number with metastatic disease[b]	WB-MRI[c]	Standard staging pathway	Difference: WB-MRI[c] - Standard	Number without metastatic disease[b]	WB-MRI[c]	Standard staging pathway	Difference: WB-MRI[c] - Standard
WB-MRI alone	68	70 (59 to 80)	63 (51 to 74)	7 (-3 to 18)	231	86 (81 to 90)	94 (90 to 96)	-8 (-13 to -3)

[a] equivocal results considered positive for colonic tumours and negative for rectal tumours

[b] Patients by consensus reference standard

[c] WB-MRI as a single standalone staging investigation

Appendix Table 6

Per-patient agreement for tumour T stage- WB-MRI staging pathways versus standard staging pathways against the consensus reference standard

T Stage	Patient number[a]	WB-MRI staging pathway[b,c] (n, %)	Standard pathway (n,%)[c]	Difference (95% CI)
T1	11	3 (27)	3 (27)	0 (-19 to 19)
T2	52	37 (71)	29 (56)	15 (2 to 29)
T3	174	100 (58)	120 (69)	-11 (-20 to -3)
T4	58	19 (33)	25 (43)	-10 (-20 to -1)
Overall T stage	295	159 (54)	177 (60)	-6 (-12 to 0)

[a]4 patients missing data

[b]WB-MRI plus additional generated tests

[c]11 and 6 patients staged Tx stage by WB-MRI and standard staging pathways respectively

Appendix Table 7

Per-patient agreement for tumour N stage- WB-MRI staging pathways versus standard staging pathways against the consensus reference standard

N Stage	Patient number[a]	WB-MRI staging pathway[b] (n, %)	Standard pathway (n,%)	Difference
N0	132	80 (60)	81 (61)	-1<br>(-9 to 7)
N1	88	45 (51)	45 (51)	0<br>(-10 to 10)
N2	74	46 (62)	40 (54)	8<br>(-3 to 19)
Overall N stage	294	171 (58)	166 (56)	2<br>(-4 to 7)

[a]5 patients missing data
[b]WB-MRI plus additional generated tests

Appendix table 8

Agreement between treatment decisions based on the WB-MRI and standard staging pathways and the retrospective 12-month consensus panel optimal treatment decision

	Total [a] N	WB-MRI staging pathway[b]		Standard staging pathway		Difference agreement WB-MRI[b] – Standard % (CI 95%)
		Agreement n (%)	Disagreement n (%)	Agreement n (%)	Disagreement n (%)	
Colorectal cancer						
All patients	296	201 (68)	95 (32)	201 (68)	95 (32)	0 (-3 to 3)
Colon cancer						
All patients	168	120 (71)	48 (29)	119 (70)	49 (30)	1 (-2 to 3)
Patients with metastatic disease	33	18 (55)	15 (45)	17 (52)	16 (48)	3 (-5 to 11)
Patients without metastatic disease	135	102 (76)	33 (24)	102 (76)	33 (24)	0 (-3 to 3)
Rectal cancer						
All patients	128	81 (63)	47 (37)	82 (64)	46 (36)	-1 (-6 to 5)
Patients with metastatic disease	32	11 (34)	21 (66)	11 (34)	21 (66)	0 (-7 to 7)
Patients without metastatic disease	96	70 (73)	26 (27)	71 (74)	25 (26)	-1 (-8 to 6)

[a]3 patients missing at least one type of patient treatment decision
[b] WB-MRI plus additional generated tests

Appendix Table 9

Investigations performed as part of the standard staging pathway

Test	Number of tests (%)[a]
CT chest, abdomen and pelvis	243 (81)
MRI pelvis/rectum [b]	120 (40)
CT chest	44 (15)
CT abdomen and pelvis	27 (9)
MRI liver	35 (12)
CT colonography	10 (3)
PET/CT	43 (14)
X-Ray [c]	9 (3)
Ultrasound [d]	12 (4)
Bone scan	2 (1)
CT liver	3 (1)
MRI abdomen and pelvis	4 (1)
Rectal ultrasound	4 (1)
Other [e]	2 (1)

[a] Patients may undergo more than one imaging test

[b] One patient underwent two MRI pelvis/rectum examinations

[c] Two patients had two X-rays (abdominal and chest)

[d] One patient had two ultrasounds (abdominal and liver)

[e] Sigmoidoscopy and unspecified CT scan

Appendix Table 10
Additional Investigations generated by WB-MRI

Test	Number of tests (%)[a]
CT chest, abdomen and pelvis	5 (2)
MRI pelvis/rectum	6 (2)
CT chest	1 (1)
CT abdomen and pelvis	0 (0)
MRI liver	0 (0)
CT colonography	0 (0)
PET/CT	0 (0)
X-Ray	0 (0)
Ultrasound	2 (1)
Bone scan	1 (1)
CT liver	1 (1)
MRI abdomen and pelvis	0 (0)
Rectal ultrasound	0 (0)
US guided biopsy	1 (1)
Other [b]	4 (1)

[a] Patients may undergo more than one imaging test
[b] one MRI spine & three MRI brain (two in the same patient)

Appendix Table 11
Number of tests required to complete staging according to the staging pathway

	WB-MRI staging pathway[a] (median test number, 95% CI)	Standard staging pathway days (median test number, 95% CI)	Difference WB-MRI[a] – Standard (95% CI)
All patients	1 (1 to 1)	2 (2 to 2)	-1 (-1 to -1)
Patients with metastatic disease	1 (1 to 1)	2 (2 to 3)	-1 (-2 to -1)
Patients without metastatic disease	1 (1 to 1)	2 (2 to 2)	-1 (-1 to -1)

[a] WB-MRI plus additional generated tests

Appendix Table 12
Time to complete staging according to staging pathway (95% CI)

	WB-MRI staging pathway[a] (days, 95% CI)	Standard staging pathway days, 95% CI)	Difference WB-MRI[a] – Standard (95% CI)
All patients	8 (6 to 9)	13 (11 to 15)	-5 (-7 to -3)
Patients with metastatic disease	8 (6 to 11)	18 (12 to 25)	-10 (-17 to -3)
Patients without metastatic disease	8 (6 to 9)	12 (11 to 14)	-4 (-7 to -2)

[a] WB-MRI plus additional generated tests

Appendix Table 13
Time to complete staging according to staging pathway-interquartile range

	WB-MRI staging pathway (median days, IQR)	Standard staging pathway (median days, IQR)
All patients	8 (4 to 13)	13 (8 to 25)

Appendix Table 14
Mean per patient staging cost according to staging pathway

	Frequency (number)			Mean cost per patient (£) (95% confidence limits)	
**Test**	**Conventional imaging**	**WB-MRI**	**Unit cost (£)**	**Conventional imaging**	**WB-MRI**
WBMRI	0	299	207	0 (0, 0)	207 (207, 207)
CT chest, abdomen and pelvis	243	5	123	100 (94, 105)	2 (0, 4)
MRI pelvis/rectum	120	6	139	56 (48, 64)	3 (1, 5)
CT chest	44	1	97	14 (10, 18)	0 (0, 1)
PET CT	43	0	484	70 (51, 88)	0 (0, 0)
MRI liver	35	0	180	21 (15, 28)	0 (0, 0)
CT abdomen and pelvis	27	0	112	10 (6, 14)	0 (0, 0)
Ultrasound	12	2	52	2 (1, 3)	0 (0, 1)
CT colonography	10	0	123	4 (2, 7)	0 (0, 0)
X Ray	9	0	19	1 (0, 1)	0 (0, 0)
Rectal ultrasound	4	0	52	1 (0, 2)	0 (0, 0)
CT liver	3	1	97	1 (0, 2)	0 (0, 1)
MRI abdomen and pelvis	4	0	207	3 (0, 5)	0 (0, 0)
Bone scan	2	1	292	2 (0, 5)	1 (0, 3)
MRI brain	0	3	180	0 (0, 0)	2 (0, 4)
CT scan – other	1	0	123	0 (0, 1)	0 (0, 0)
Sigmoidoscopy	1	0	169	1 (0, 2)	0 (0, 0)
US guided biopsy	0	1	92	0 (0, 0)	0 (0, 1)
MRI spine	0	1	180	0 (0, 0)	1 (0, 2)
Total				285 (260, 310)	216 (211, 221)

Patients may undergo more than one of the same imaging test. Tests ranked by the most frequent across both arms. All costs are 2016/17 UK£. 95% confidence limits derived from 1000 bootstrapped replications of the mean
